# An international delphi survey for the definition of the variables for the development of new classification criteria for periodic fever aphtous stomatitis pharingitis cervical adenitis (PFAPA)

**DOI:** 10.1186/s12969-018-0246-9

**Published:** 2018-04-18

**Authors:** Federica Vanoni, Silvia Federici, Jordi Antón, Karyl S. Barron, Paul Brogan, Fabrizio De Benedetti, Fatma Dedeoglu, Erkan Demirkaya, Veronique Hentgen, Tilmann Kallinich, Ronald Laxer, Ricardo Russo, Natasa Toplak, Yosef Uziel, Alberto Martini, Nicolino Ruperto, Marco Gattorno, Michael Hofer, for Eurofever and the Paediatric Rheumatology International Trials Organisation (PRINTO)

**Affiliations:** 1 Department of Pediatrics, Ospedale San Giovanni, 6500 Bellinzona, Switzerland; 2 Unité Romande d’Immuno-rhumatologie Pédiatrique, CHUV, University of Lausanne, Lausanne, and HUG, Geneva, Switzerland; 3 Istituto Giannina Gaslini, Clinica Pediatrica e Reumatologia, PRINTO, Genoa, Italy; 4 Pediatric Rheumatology, Hospital Sant Joan de Déu, Universitat de Barcelona, Esplugues de Llobregat, Barcelona Spain; 5 NIH-NIAID, Division of Intramural Research, Bethesda, MD USA; 6 Department of Infection, Inflammation and Rheumatology, Great Ormond St Hospital, London, UK; 7 IRCCS Ospedale Pediatrico Bambino Gesù, Division of Rheumatology, Rome, Italy; 8 Division of Immunology, Rheumatology Program, Harvard Medical School Boston, Boston Children’s Hospital, Boston, MA USA; 9 Division of Paediatric Rheumatology, Western University and Children’s Hospital LHSC, London, ON Canada; 10 Hôpital A Mignot - Centre Hospitalier de Versailles, CEREMAIA, French reference center for autoinflammatory diseases and inflammatory amyloidosis, Le Chesnay (Paris), France; 11 Pediatric Pneumology and Immunology, Charité Universitätsmedizin Berlin, Berlin, Germany; 12 Departments of Pediatrics and Medicine, University of Toronto and The Hospital for Sick Children, Toronto, ON Canada; 13 Servicio de Inmunología y Reumatología, Hospital de Pediatría Juan P. Garrahan, Buenos Aires, Argentina; 14 Department of Allergology, Rheumatology and Clinical Immunology, University Children’s Hospital, University Medical Center Ljubljana, Ljubljana, Slovenia; 15 Meir Medical Centre, Pediatric Rheumatology Unit, Department of Pediatrics, Kfar Saba and Sackler School of Medicine, Tel Aviv University, Tel Aviv-Yafo, Israel; 16 Istituto Giannina Gaslini, Direzione Scientifica, Genoa, Italy

**Keywords:** Autoinflammatory diseases, Monogenic periodic fever, PFAPA

## Abstract

**Background:**

Diagnosis of Periodic fever, aphthous stomatitis, pharyngitis and cervical adenitis (PFAPA) is currently based on a set of criteria proposed in 1999 modified from Marshall’s criteria. Nevertheless no validated evidence based set of classification criteria for PFAPA has been established so far. The aim of this study was to identify candidate classification criteria PFAPA syndrome using international consensus formation through a Delphi questionnaire survey.

**Methods:**

A first open-ended questionnaire was sent to adult and pediatric clinicians/researchers, asking to identify the variables thought most likely to be helpful and relevant for the diagnosis of PFAPA. In a second survey, respondents were asked to select, from the list of variables coming from the first survey, the 10 features that they felt were most important, and to rank them in descending order from most important to least important.

**Results:**

The response rate to the first and second Delphi was respectively 109/124 (88%) and 141/162 (87%). The number of participants that completed the first and second Delphi was 69/124 (56%) and 110/162 (68%). From the first Delphi we obtained a list of 92 variables, of which 62 were selected in the second Delphi. Variables reaching the top five position of the rank were regular periodicity, aphthous stomatitis, response to corticosteroids, cervical adenitis, and well-being between flares.

**Conclusion:**

Our process led to identification of features that were felt to be the most important as candidate classification criteria for PFAPA by a large sample of international rheumatologists. The performance of these items will be tested further in the next phase of the study, through analysis of real patient data.

**Electronic supplementary material:**

The online version of this article (10.1186/s12969-018-0246-9) contains supplementary material, which is available to authorized users.

## Background

Periodic fever, aphthous stomatitis, pharyngitis and cervical adenitis (PFAPA) is a recurrent fever syndrome that was first described in 1987 [1]. The syndrome is characterized by episodes of fever lasting 3–6 days with a regular recurrence (every 3–8 weeks), associated with at least one additional clinical feature among aphthous stomatitis, cervical adenitis, and pharyngitis [2]. PFAPA is not a well-defined disease and there are no specific confirmatory laboratory or genetic tests, which differs from the hereditary periodic fever (HPF) syndromes. Currently the diagnosis of PFAPA is based on modified Marshall’s criteria [2], but the results of a recent survey emphasize the poor adherence of most physicians to these criteria in their clinical practice [3]. Despite a good sensitivity it has been demonstrated that a significant number of patients with monogenic periodic fevers (familial mediterranean fever (FMF), Tumor Necrosis Factor (TNF) receptor-associated periodic syndrome (TRAPS), and Mevalonate Kinase Deficiency (MKD)) also meet the diagnostic criteria for PFAPA syndrome, highlighting the poor specificity of the diagnostic criteria [4, 5].

At variance with diagnostic criteria that should reflect the heterogeneity of a disease in order to identify as many people with the condition as possible, with high sensitivity, the main purpose of classification criteria is to standardize clinical definitions to be used in clinical and pathogenic studies. For classification criteria, very high specificity is required, whereas for diagnostic criteria both specificity and sensitivity need to be close to 100%, which is difficult to achieve [6].

We report herein the results of an international project aimed to identify the most important variables for the proper classification of PFAPA patients. This is the first step of a process aimed to develop new classification criteria for PFAPA, through consensus formation technique and data validation in the large dataset of the Eurofever Registry [7].

## Methods

For the development of the classification criteria for PFAPA we used a multistep approach. The first step (Delphi) which attempted to identify the most important variables for the proper classification of PFAPA is described in this manuscript.

Delphi Technique [8, 9] is a well-known consensus formation method derived from the social sciences which involves the attainment of consensus among a large group of people through a series of surveys. While the first survey is typically open ended, the following surveys are conceived based on the results of the prior ones. Multiple international electronic surveys have been conducted via the secured web based system of the Paediatric Rheumatology INternational Trials Organization [10, 11] (PRINTO).

The first Delphi questionnaire was sent by e-mail to all centers belonging to the PRINTO network that enrolled at least one patient in the Eurofever registry [7]. The surveys involved both adult and pediatric clinicians/researchers worldwide. Participants were asked to identify the variables thought most likely to be helpful and relevant for the diagnosis of PFAPA in current clinical practice and in research setting. In the first survey participants were asked to report, in an open fashion, all measures they thought to be relevant for PFAPA based on their clinical or research expertise. The question was: “Please list the variables (as many as you like) that you are currently using in your everyday clinical practice or you consider to be the most useful for the diagnosis of PFAPA. Variables to be included can be of any type: i.e. clinical features, laboratory tests, genetic analysis etc.”

From the first survey we obtained a list of clinical and laboratory variables which were checked for redundancy and categorized into 5 domains. For each category, the variables were listed in in alphabetical order with no indication about the frequency of response for each item. Additionally the list was updated with other variables derived from a literature search performed by FV, SF and MG.

The second Delphi questionnaire, based on the result of the first one, was sent to all participants from the first survey. In addition 43 North American clinicians/researchers working in the field of autoinflammation and members of Pediatric Rheumatology Collaborative Study Group (PRCSG) and Childhood Arthritis and Rheumatology research Alliance (CARRA), were also involved. Participants were first asked to choose, among the variables listed, the 10 they consider as the most important for the classification of PFAPA. In a second step, they were asked to rank the previously selected items by assigning them a score from 10 to 1 where 10 was for the most important item and 1 for the least important. They could use each rank only once, even though they considered some features were equally important. At the end of the questionnaire, the participants could add any feature missing from the list that they considered as relevant. The question was: “Please choose, from the above mentioned list of items, the 10 top variables for the classification of PFAPA and rank them in order of importance.”

At least two reminders e-mail were sent to all investigators who had not replied to the first or second survey.

The sum of ranks, frequency of citation and medium score for each variable were calculated.

Variables falling in the 3rd quartile, considering the total score obtained will be included in the subsequent phases of the development of classification criteria in which the ability of different set of criteria to classify individual patients as having PFAPA will be assessed. The best sets of criteria identified will be discussed in a face-to-face consensus conference.

## Results

The first survey was sent to 124 participants coming from 63 countries.

**Fig. 1:**
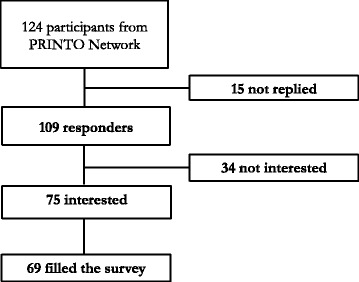
Participants to the first survey. Summarize the participation rate to the first survey, specifying number of recipients that not replied, number of recipients interested in the survey and number of recipients that filled the survey

One hundred nine recipients responded (88%): 75 (70%) participants responded to be interested in the survey, 69 (56%) completed and confirmed it, 34 (27%) responded not to be interested, 15 (12%) did not reply (Fig. 1).

We obtained a list of 92 variables (Additional file 1: Table S1 and S2) subsequently classified into 5 mutually exclusive categories: 11 for characteristics of fever episodes, 41 for signs and symptoms, 12 for history, 30 for laboratory parameters, 4 for others tests. Criteria from the literature review were included in the list [2]. The five most cited variables were periodic fever, cervical adenitis, aphthous stomatitis, pharyngo-tonsillitis and abdominal pain. The exclusions of inherited monogenic periodic fever and the response to corticosteroids were cited by 24 and 43% of participants respectively. Several participants cited as important the absence of specific clinical manifestations i.e. absence of serositis or absence of diarrhea.

The second survey was sent to 162 physicians, 119 from PRINTO and 43 from CARRA/PRCSG.

**Fig. 2:**
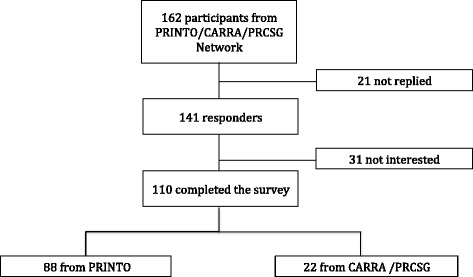
Participants to the second survey. Summarize the participation rate to the second survey, specifying number of recipients that not replied, number of recipients interested in the survey and number of recipients that filled the survey, divided between PRINTO and CARRA networks

The overall rate of response was 87% (141/162): 110 (68%) experts completed the survey 88 from Eurofever/PRINTO network and 22 from CARRA/PRCSG, 31 (19%) responded not to be interested, 21 (13%) didn’t respond (Fig. 2).

At the end of the second Delphi survey we obtained a list of 62 variables. For each variable the total score (given by the sum of the score attributed by the participants citing that variable) was calculated.

For the subsequent statistical analysis we selected the first 17 variables falling in the 3rd quartile considering the total score obtained. The ranking including frequencies of selection and mean score is shown in Table 1.

**Table 1:** Variables rank limited to the 3rd quartile

Rank	Variable	Score	Frequence	Mean score
1	Regular periodicity	436	56	7.8
2	Aphtous stomatitis	431	77	5.6
3	Response to steroid	401	66	6.1
4	Cervical adenitis	368	72	5.1
5	Well-being between flares	299	57	5.2
6	Pharyngitis (exudative or not)	288	47	6.1
7	Increase of acute phase reactants and serum amyloid A during fever episodes	271	44	6.2
8	Normal growth/development	236	51	4.6
9	Pharingotonsillitis	228	35	6.5
10	Periodic fever 3–5 days	202	24	8.4
11	Periodic fever 3–6 days	202	23	8.8
12	Self-limiting episodes	183	35	5.2
13	Response to tonsillectomy	182	33	5.5
14	Improvement with age	160	40	4.0
15	Exclusion cyclic neutropenia/immunodeficiency	150	34	4.4
16	Normalization of acute phase reactants in well-being	146	33	4.4
17	Recurrence every 3–6 weeks	145	21	6.9

The highest score of 436 was reached by “regular periodicity” whereas the most selected clinical variable was aphthous stomatitis chosen by 77 participants.

None of the participants added any variables to the list proposed, suggesting that participants agreed that the items coming from the first survey were exhaustive.

All cardinal clinical signs (aphthous stomatitis, cervical adenitis and pharyngitis) were included in the third quartile at the second, fourth and sixtieth rank respectively. Regular periodicity reached the highest score, highlighting the importance of regularity of episodes in the diagnosis of PFAPA. Response to corticosteroids and response to tonsillectomy were also present. Concerning the duration of fever episodes two items reached the same score: duration of 3–5 days and duration of 3–6 days.

Others variables with a high rank were raised acute phase reactants during fever episodes and their normalization between episodes, wellbeing between episodes, normal growth and development and improvement with age.

Other variables like gastrointestinal/musculoskeletal symptoms, headache and positive family history were cited, but didn’t reach a sufficient score to be include in the 3rd quartile (Additional file 1: Table S1 and S2).

Notably, age at onset < 5 years, which is present in the modified Marshall’s criteria reached the 22th rank only.

## Discussion

This is the first Delphi survey for the identification of candidate variables for a new set of classification criteria for PFAPA. Using the Delphi technique process involving a large number of clinicians and researchers worldwide dealing with this condition, we obtained a list of variables ranked in order of importance considered as important for the identification of PFAPA patients.

The good rate of response to the Delphi underlined the interest that clinicians and researchers involved in autoinflammation have in this area. Globally, the results of the Delphi reflect the elements that clinicians take into account to identify PFAPA patients.

Participants confirmed the importance of most of the variables already included in the modified Marshall’s diagnostic criteria such as regular periodicity, aphthous stomatitis, cervical adenitis, pharyngitis and wellbeing between flares and normal growth and development.

The variable “Age at onset < 5 years” reached the 22th rank, suggesting that currently this features is no longer considered such important. This may be due to the fact that in the last few years it has been observed that PFAPA onset may occur after 5 years [12] and to date several cases of adult onset PFAPA have been described [13]. This finding was confirmed in a recent study in which was demonstrated that most physicians do not require an age at onset < 5 years for diagnosis of PFAPA [3].

The exclusion of cyclic neutropenia is cited in the list together with exclusion of immunodeficiency. Cyclic neutropenia is an important differential diagnoses for PFAPA, due to its strict periodicity. This condition generally begins within the first year of life and is characterized by a cyclic reduction (every 3 weeks) of the neutrophil count causing febrile attacks due to infections. For this reason a careful evaluation of cell blood count during the first day of fever attacks may be useful in the differential diagnosis.

Items associated to the exclusion of confounding infectious conditions, such as “Absence of response to antibiotics”, “Negative throat swab”, “Absence of concomitant respiratory symptoms” were cited by the clinicians but reached the 21st, 25th and 27th rank only, respectively. The fact that these three items reached a low score may be due to the fact that for physicians involved in management of PFAPA the exclusion of the other causes of recurrent fever (mainly upper respiratory airways recurrent infections) is implicit when they consider a diagnosis of PFAPA in the everyday clinical practice.

Less typical manifestations such headache, gastrointestinal or musculoskeletal symptoms were included by some clinicians, but did not reach the third quartile (Additional file 1: Table S1 and S2). It is known that, even if these features might be occasionally presents in PFAPA subjects [12], the presence of abdominal pain and diarrhea, are much more associated with the probability to carry relevant mutations in *MVK*, *TNFRSF1A* and *MEFV* genes associated with mevalonate kinase deficiency, TRAPS and familial Mediterranean fever, respectively [14]. Therefore, the absence of these symptoms may strengthen the suspicion of PFAPA.

Both the elevation of acute phase reactants during fever episodes and their normalization in the periods between episodes were indicated by participants and reached the third quartile. An increase of the inflammatory parameters during episodes is a mandatory element to point out a possible autoinflammatory periodic fever [15], but does not help in the discrimination of PFAPA from other monogenic periodic fevers in clinical practice. The same is also valid for the normalization of inflammatory parameters between fever episodes, even if in some conditions (FMF, cryopyrin associated periodic syndromes) sub-clinical systemic inflammation can be detected.

The lack of pathognomonic test for PFAPA (i.e genetic test or metabolic analysis available for other monogenic periodic fevers) might increase the relevance of the response to therapy (corticosteroid on demand or tonsillectomy) in this condition. Indeed, both response to corticosteroids and tonsillectomy were included in the third quartile of the scores. Response to corticosteroids on demand in particular reached the third rank. This element was not considered in the previous criteria but reflect an important element adopted by clinicians in routine clinical practice. However it should be noted that the same good response to corticosteroid on demand has been reported in some monogenic periodic fevers, like MKD and TRAPS [16]. It is questionable whether the response to tonsillectomy is a good element to support the diagnosis of PFAPA, since response to tonsillectomy is also found in non-PFAPA recurrent tonsillitis. Moreover, it is critical to confirm the diagnosis before a surgical procedure that furthermore is not always necessary in PFAPA patients.

The exclusion of other monogenic periodic fevers, mainly MKD is a crucial step for the classification of PFAPA [4]. According to the clinicians involved a “Negative genetic test for monogenic autoinflammatory diseases” resulted at the 18th rank, falling out the 3rd quartile. It is conceivable that, especially in typical cases the genetic analysis of genes associated to HPF is not mandatory for the proper classification of a PFAPA patient. On the other hand, the identification of some variants of unknown significance in genes responsible for other inflammatory conditions (i.e R202Q or E148Q for *MEFV*, Q703K for *NLRP3* gene, P46L or R92Q for *TNFRSF1A* gene) should not exclude the classification of PFAPA in a patient with a typical clinical phenotype [12]. It seems therefore crucial to elaborate clinical classification criteria able to discriminate PFAPA from other autoinflammatory periodic fever with an high sensitivity and specificity. Along these lines it is important to highlight that among the variables cited in the first Delphi, many participants indicated also the absence of some manifestations rather typical for monogenic periodic fevers, such as serositis, arthritis, diarrhea, eye involvement (Additional file 1: Table S1 and S2). This is an interesting finding that underlined the fact that sometimes even the absence of some symptoms may have a relevant place in the classification of a given disease, as recently shown by the preliminary evidence-based criteria for monogenic HPF developed from the Eurofever registry [17].

A possible limitation of this study is that specialists possibly involved in the management of PFAPA patients (infectious diseases specialists or oto-rhino-laryngologists) were not part of this process. Nonetheless, the notion that recurrent inflammatory fever episodes characteristics of PFAPA should be carefully distinguished from other possible causes of recurrent fever (infections, immunodeficiency, neoplasms) is rather consolidated in the pediatric community [18]. Since, the major limitation of the ongoing PFAPA criteria is related to their low accuracy in differentiating this condition from monogenic periodic fevers (MKD, TRAS and FMF) [4], our main goal was to involve a large number of clinicians with expertise in the identification of all inflammatory causes of periodic/recurrent fevers, in order to identify clinical variables that would be able to distinguish PFAPA on clinical grounds, without the need to perform a molecular tests.

## Conclusions

In conclusion, this study represents the largest Delphi survey among clinicians and researchers dealing with PFAPA and other autoinflammatory periodic fevers. The process allowed the identification of those features that are considered by clinicians the most important as candidate variables to be included in a new set of evidence-based classification criteria for PFAPA. The performance of these items will be tested further in the next phase of the study, through analysis of real patient data.

## Additional file

Additional file 1: **Table S1.** Variables coming from the first survey. **Table S2.** Variable rank from the second Delphi. (DOCX 662 kb)

## Abbreviation

FMF: Familial mediterranean fever
HPF: Hereditary periodic fevers
MKD: Mevalonate kinase deficiency
PFAPA: Periodic fever aphtous stomatitis pharingitis cervical adenitis
TNF: Tumor necrosis factor
TRAPS: Tumor necrosis factor associated periodic syndrome
