# Supplementary material for: An international delphi survey for the definition of the variables for the development of new classification criteria for periodic fever aphtous stomatitis pharingitis cervical adenitis (PFAPA)
Source: Pediatr Rheumatol Online J. 2018 Apr 18;16:27. doi: 10.1186/s12969-018-0246-9 (PMC5907175; doi:10.1186/s12969-018-0246-9)
Supplement: Supplementary file 1 — Table S1. Variables coming from the first survey. Table S2. Variable rank from the second Delphi. (DOCX 662 kb) [file 12969_2018_246_MOESM1_ESM.docx]

Table S1. Variables coming from the first survey

Table S2. Variable rank from the second Delphi
